# Repetitive Transcranial Magnetic Stimulation for the Treatment of Spinal Cord Injury: Current Status and Perspective

**DOI:** 10.3390/ijms26020825

**Published:** 2025-01-19

**Authors:** Shu Fan, Wei Wang, Xiaolong Zheng

**Affiliations:** 1 Department of Neurology, Tongji Hospital, Tongji Medical College, Huazhong University of Science and Technology, Wuhan 430030, China; shufan@hust.edu.cn; 2 Hubei Key Laboratory of Neural Injury and Functional Reconstruction, Huazhong University of Science and Technology, Wuhan 430030, China; 3 Key Laboratory of Neurological Diseases of Chinese Ministry of Education, the School of Basic Medicine, Tongji Medical College, Huazhong University of Science and Technology, Wuhan 430030, China

**Keywords:** spinal cord injury, transcranial magnetic stimulation, noninvasive brain stimulation, neuromodulation, neuroplasticity, motor dysfunction, pain, spasticity

## Abstract

Spinal cord injury (SCI) can lead to devastating dysfunctions and complications, significantly impacting patients’ quality of life and aggravating the burden of disease. Since the main pathological mechanism of SCI is the disruption of neuronal circuits, the primary therapeutic strategy for SCI involves reconstructing and activating circuits to restore neural signal transmission. Repetitive transcranial magnetic stimulation (rTMS), a noninvasive brain stimulation technique, can modulate the function or state of the nervous system by pulsed magnetic fields. Here, we discuss the basic principles and potential mechanisms of rTMS for treating SCI, including promoting the reconstruction of damaged circuits in the spinal cord, activating reorganization of the cerebral cortex and circuits, modulating the balance of inputs to motoneurons, improving the microenvironment and intrinsic regeneration ability in SCI. Based on these mechanisms, we provide an overview of the therapeutic effects of rTMS in SCI patients with motor dysfunction, spasticity and neuropathic pain. We also discuss the challenges and prospectives of rTMS, especially the potential of combination therapy of rTMS and neural progenitor cell transplantation, and the synergistic effects on promoting regeneration, relay formation and functional connectivity. This review is expected to offer a relatively comprehensive understanding and new perspectives of rTMS for SCI treatment.

## 1. Introduction

Spinal cord injury (SCI) is a devastating neural trauma that results in neurological dysfunction and severe complications, including neuropathic pain, spasticity, and autonomic dysfunction. Over the past 30 years, the incidence of SCI and the burden of disease have increased significantly [1,2]. In 2019, 900,000 new cases and 20.6 million prevalent cases were reported [3]. Owing to the leading causes of SCI, falls and road injuries [1], as well as the growth and aging of the population, the burden of SCI remains a concern. Thus, novel potential interventions and treatments for SCI are desperately needed.

Transcranial magnetic stimulation (TMS), a noninvasive brain stimulation technique, can modulate neurons locally and distantly by generating a pulsed magnetic field with a copper coil, thus inducing secondary electrical currents [4]. Repetitive transcranial magnetic stimulation (rTMS), one of the most common TMS stimulation protocols, can produce effects that exceed those of stimulation alone [5]. With progress in the understanding of SCI pathological processes, neuromodulation, and neuroplasticity, the mechanisms of rTMS have received increasing attention. To repair the lesion and restore the function of the spinal cord, the reconstruction and activation of neural circuits are critical and necessary. According to current theories, neuroplasticity serves as the fundamental basis of recovery after SCI, representing the intrinsic potential of the nervous system to undergo functional and structural changes [6]. In general, it is believed that neuroplasticity might be activity-dependent [6,7,8], which indicates that external stimuli of the nervous system, such as rTMS, might induce neuroplasticity and improve recovery after SCI [9,10].

rTMS can play a role at various levels and in multiple forms. At the level of the spinal cord with the lesion, rTMS can activate the spared circuits and reconstitute the damaged circuits [11,12]; at the level of the network above the SCI, rTMS can induce reorganization of the cerebral cortex and corticospinal circuits [13,14,15]; at the level of motoneurons downstream of the spinal cord, rTMS can modulate the balance of excitatory and inhibitory inputs to the motoneurons [16,17]; and at the level of the molecules, cells and microenvironment, rTMS might inhibit the activation of astrocytes [18,19], augment the intrinsic regeneration ability of neurons in the spinal cord [12,19,20,21], and protect the neurons from apoptosis or necrosis [22,23,24].

Furthermore, rTMS has been utilized in a large number of clinical trials for SCI, demonstrating its therapeutic effectiveness in various aspects, including increasing corticospinal transmission, enhancing voluntary motor output, improving motor function, alleviating spasticity and neuropathic pain, and relieving autonomic dysfunction. Many of these studies have achieved positive results. In recent decades, many novel protocols and therapies have been developed, such as intermittent theta-burst stimulation (iTBS) [25,26] and paired associative stimulation (PAS), which combine TMS with peripheral nerve stimulation (PNS), transcutaneous spinal cord stimulation (tSCS), or precise root stimulation [27,28]. rTMS can also be used as an adjuvant therapy alongside other interventions, such as rehabilitation and biological strategies [2]. These innovative approaches represent potential directions for the development of rTMS for the treatment of SCI. For example, among these alternative combination therapies, the transplantation of neural progenitor cells (NPCs) is a potential candidate because of its synergistic effects on promoting regeneration [29,30] and functional connectivity formation [31,32] with rTMS.

In this review, we introduce the basic concepts and perspectives of TMS and focus on the latest research progress regarding the potential mechanisms and therapeutic effects of rTMS in the treatment of SCI. We also present the current challenges and prospectives of rTMS in SCI treatment, expecting to provide a relatively comprehensive understanding and a new outlook on rTMS in the treatment of SCI.

## 2. Basic Principles of rTMS

**Figure 1:**
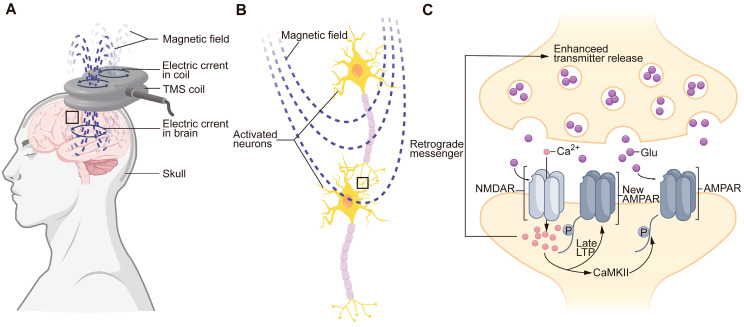
Basic principles of TMS: (**A**) TMS is a noninvasive brain stimulation technique that can induce pulsed magnetic fields and second electrical currents in the brain via a copper coil. The boxed area in (**A**) is magnified in (**B**). (**B**) rTMS activates neurons and induces synaptic plasticity. The boxed area in (**B**) is magnified in (**C**). (**C**) LTD-like effect of rTMS. When the postsynaptic glutamate receptor (NMDAR) is activated, Ca^2+^ can flow into postsynaptic neurons, which can subsequently induce phosphorylation and increase the number of AMPARs, major postsynaptic glutamate receptors at excitatory synapses that mediate fast neurotransmission and synaptic plasticity. rTMS can also increase the number of transmitters released by presynaptic neurons. TMS, transcranial magnetic stimulation; NMDARs, NMDA receptors; AMPARs, AMPA receptors; Glu, glutamate; LTP, long-term potentiation.

TMS is a noninvasive brain stimulation technique that uses a pulsed magnetic field generated by a copper coil to induce secondary electrical currents in the brain, thereby modulating the function or state of the nervous system [4,33] (Figure 1A). Compared with transcranial electrical stimulation, TMS can effectively stimulate the brain through the intact scalp and skull without causing the pain of scalp irritation and scalp muscle contraction [34]. The common patterns of TMS include single-pulse TMS, paired-pulse stimulation, rTMS and PAS. rTMS has been extensively studied and applied in clinical trials and therapies because of the increase in after-effects exceeding the stimulus duration [4,34].

The intensity of rTMS decreases rapidly with increasing distance between the coil and cortical surface, and it is generally assumed that rTMS is limited to the cortex and superficial subcortical regions [4]. The effect of rTMS varies with many factors, including the shape and orientation of the coil, the differentiation of neuron subsets, and the underlying structure and activity of the cortex [35,36]. Additionally, the stimulation protocol (frequency, stimulus duration, intensity, pulse shape, etc.) also contributes to the variability in the effects of rTMS [37].

In recent years, research on rTMS has gradually increased. In response to rTMS, the human nervous system can undergo functional and structural changes. rTMS can induce immediate effects lasting a few seconds to minutes by directly activating or inhibiting cortical neurons. This may be associated with changes in ionic balance, electrical capacitive effects, and feedback from the targets of the stimulated neurons [4,38]. Moreover, rTMS can also induce after-effects that exceed the duration of stimulation. However, the underlying mechanisms require further investigation.

According to current theories, it is supposed that the after-effects of rTMS are attributed primarily to synaptic plasticity—long-term potentiation (LTP) and long-term depression (LTD)—as they share several fundamental characteristics supporting this hypothesis (Figure 1B,C). First, the effect of rTMS is frequency dependent, similar to LTP/LTD—a brief period of high-frequency stimulation can activate neurons exceeding the duration of stimulation, whereas low-frequency stimulation induces inhibitory after-effect [39,40,41]. It can also be influenced by the underlying cortical state, including the cognitive state, oscillatory state, and recent history of the brain state [42]. Second, similar to LTP, in the early stage after rTMS (lasting 30 to 60 min), the effect is caused by increasing the number of transmitters released by presynaptic neurons [43,44,45] and enhancing the response of postsynaptic neurons to transmitters (e.g., by increasing the number of N-methyl-D-aspartate receptors (NMDARs) and AMPA receptors (AMPARs)) [46,47,48,49,50].

Subthreshold rTMS can drive structural synaptic plasticity, which is a key mechanism of subthreshold rTMS-induced neuroplasticity. It altered the rate of dendritic spine loss and gain in the mouse motor cortex, highlighting the ability to alter synaptic connectivity in the brain [50,51].

However, synaptic plasticity is not the only potential mechanism underlying rTMS. The long-term effects of rTMS, which can last from days to a few weeks, may be due mainly to the regulation of gene expression and protein synthesis, which increase the intrinsic growth potential of neurons, augment neuroplasticity, and change the microenvironment of the injured spinal cord.

## 3. Research Progress on the Mechanisms of rTMS in SCI

### 3.1. rTMS Recruits Spared Circuits and Reconstitutes Damaged Circuits in the Spinal Cord

**Figure 2:**
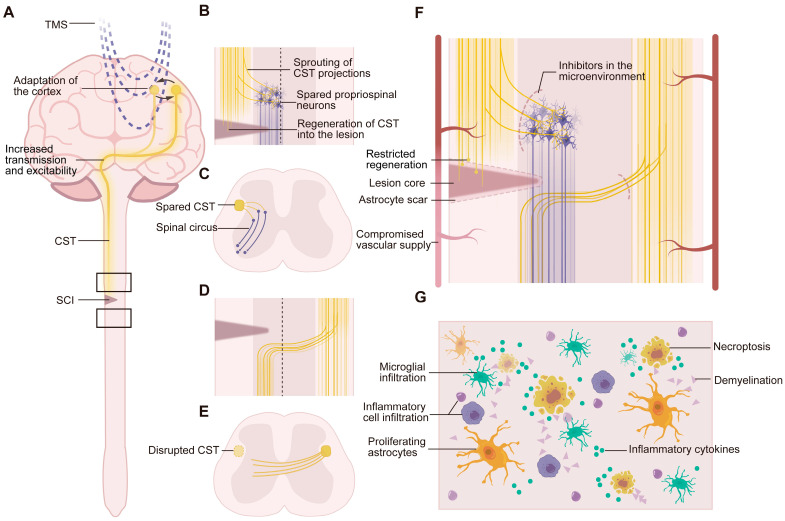
Potential mechanisms underlying the effects of rTMS for SCI (**A**) SCI and rTMS induce functional and structural adaptations in the cortex, neural circuits above the SCI site and the spinal cord. SCI, spinal cord injury; CST, corticospinal tract. (**B**), (**C**) The upper boxed area in (**A**) is magnified in (**B**) (coronal section) and (**C**) (transverse section). rTMS promotes activity-dependent sprouting and growth of residual CST projections to propriospinal neurons as relay circuits in incomplete SCI. rTMS can also enhance the regeneration of corticospinal axons into the lesion. (**D**), (**E**) The lower boxed area in (**A**) is magnified in (**D**) (coronal section) and (**E**) (transverse section). rTMS promotes the sprouting of contralateral corticospinal axons projecting to the injured side below the SCI. (**F**) rTMS improves the microenvironment, including alleviating astrocyte activation and scarring, improving the vascular supply, and reducing the number of inhibitors in the microenvironment. rTMS potentiates the intrinsic regeneration ability of neurons in the spinal cord, promoting axon regeneration, axon sprouting, and synaptic plasticity. (**G**) Some main inhibitory factors for axon regeneration and sprouting in the microenvironment, which may be improved with rTMS therapy, including astrocyte activation, microglial infiltration, inflammatory cell and cytokine infiltration, and tissue and cell debris from demyelination and necroptosis. rTMS, repetitive transcranial magnetic stimulation.

SCI can cause devastating damage to the spinal cord through initial mechanical injury and sequential cellular and biological reactions, resulting in the loss of neurons, damage to synapses, and disruption of neuronal circuits (Figure 2A). Thus, the reconstruction and activation of neural circuits are critical for functional recovery after SCI. SCI can be classified into anatomically complete SCI and anatomically incomplete SCI (iSCI) according to the severity of the injury. In iSCI, there are spared circuits that can be activated and reorganized, promoting functional recovery with axon sprouting and synapse remodeling [52]. When SCI is complete, however, restoring the signals after SCI requires axonal regeneration and synaptogenesis across the lesion [53].

rTMS, as a noninvasive stimulation of neurons, can induce output signals and access downstream circuits. A growing body of evidence indicates that rTMS can promote the activity-dependent regeneration of corticospinal tract (CST) axonal projections and the recruitment of propriospinal neurons and circuits (Figure 2B–E). Several studies have drawn conclusions indirectly on the basis of the improved outcomes of electrophysiological parameters and motor functions. A previous study recorded changes in surface electromyography (sEMG) and motor-evoked potential (MEP) signals from both the abductor pollicis brevis and tibialis anterior muscles after rTMS therapy, suggesting increased transmission to the spinal cord and muscles. These results support the hypothesis that residual efferent pathways and propriospinal neurons are important for rTMS therapy in SCI patients [11]. Another study reported that TMS combined with peripheral stimulation could increase voluntary motor output through corticospinal–motoneuronal synapses in SCI patients, as manifested by the increase in the size of MEPs elicited in the resting first dorsal interosseous muscle, which in turn improved the speed at which the nine-hole peg test (9HPT) was completed [27]. In 2023, Boato et al., however, observed robust regeneration of corticospinal axons after rTMS through neural circuit tracers in the rodent model via histology. High-frequency rTMS (HF-rTMS) reportedly improves corticospinal axon sprouting and regeneration across dorsal hemisection injuries. For unilateral pyramidotomy, HF-rTMS increased regeneration and sprouting of the contralateral CST projecting to propriospinal neurons and forming synaptic connections, which constituted new relay circuits. In both SCI models, HF-rTMS promoted motor recovery in SCI model mice [12].

Yamanaka et al. demonstrated that TMS following treatment with an antibody against repulsive guidance molecule-a (RGMa) induced CST axons to extend across the SCI into laminae VII–IX of the spinal cord and facilitated CST activity, improving the motor function of SCI primates [54,55]. They also reported that Ca^2+^/calmodulin-dependent kinase II (CaMKII) in the motor cortex, which is known as a key mediator of LTP (Figure 1C), was upregulated in mice treated with anti-RGMa antibody and rTMS sequentially compared with that in mice treated with anti-RGMa antibody alone, suggesting synergistic effects of anti-RGMa antibody and rTMS on increasing motor output in SCI [56].

### 3.2. rTMS Activates Reorganization of the Cerebral Cortex and Corticospinal Circuits Above the Site of SCI

Although SCI causes damage to partial segments of the spinal cord, the circuits above and below the SCI are preserved and can reorganize spontaneously in response to the trauma. Time-dependent, bilateral reorganization of cortical dynamics is observed in lateralized SCI NHP models associated with motor functional recovery [57], indicating the importance of the reorganization of cerebral cortex functional connectivity after SCI. A study indicated that rTMS could enhance the functional connectivity of the cortex-thalamus and multiple brain regions and activate the hypothalamic pituitary adrenal (HPA) axis in the brain, which could be a potential mechanism for alleviating neuropathic pain after SCI [22].

Importantly, neuroplasticity is believed to be activity-dependent, which implies that the adaptive circuit reorganization that is beneficial for functional recovery requires stimulus activation and guidance [7]. Some studies have concluded that rTMS, as an external stimulus, can change the cortical drive and corticospinal pathways and subsequently affect the circuits in the spinal cord [13,14,58]. It has been reported that long-term subthreshold HF-rTMS can induce practice-dependent plasticity by increasing transmission in synaptic connections and reinforcing synaptic connections in the motor cortex and between the motor cortex and spinal motoneurons [15] (Figure 2A).

Correspondingly, the lack of proper stimulus and activation for patients with SCI usually leads to maladaptive plasticity, which is one of the extensively researched mechanisms underlying neuropathic pain and spasticity [7,59]. Therefore, rTMS provides a novel method to modulate neuroplasticity to alleviate neuropathic pain and spasticity by providing the necessary stimulus and activation [59,60].

### 3.3. rTMS Modulates the Balance of Excitatory and Inhibitory Inputs to Motoneurons

Motor recovery following SCI relies on excitatory inputs to motoneurons from the spinal cord. However, hyperactivity of motoneurons can induce spasticity, in turn significantly aggravating motor dysfunction. Several studies have provided insight into the moderating effect of rTMS on the balance of excitatory and inhibitory inputs to motoneurons, thereby potentiating motor recovery and alleviating spasticity. rTMS combined with treadmill training can increase the expression of serotonin and promote the growth of serotonergic axons in the spinal cord of SCI rats [16], which is positively correlated with the sensitivity and excitability of motoneurons and motor function recovery [61,62].

In addition, several studies have demonstrated the inhibitory modulation and relief of hyperreflexia and spasticity induced by rTMS. The underlying mechanisms contributing to the increase in inhibitory inputs may involve the upregulation of potassium-chloride cotransporter-2 (KCC2) and glutamic acid decarboxylase 67 (GAD67), leading to the adjustment of chloride channels in neurons and increased synthesis of gamma-aminobutyric acid (GABA), respectively [16,17].

### 3.4. rTMS Improves the Microenvironment and Potentiates the Intrinsic Regeneration Ability of Neurons in the Spinal Cord

Although the nervous system has an intrinsic repair mechanism, the effectiveness of recovery remains insufficient and unsatisfactory. The failure of functional recovery after SCI is traditionally attributed to an inappropriate microenvironment and deficient intrinsic neuronal growth competence [53] (Figure 2F,G).

Astrocyte and microglial activation are two factors that inhibit axonal regeneration, circuit reconstruction and functional restoration. A study revealed that the elevated expression of specific microglial and astrocyte markers, Iba1 and GFAP, in the spinal cord below SCI was significantly reduced by approximately 30% after 25 Hz rTMS therapy for 8 weeks [18], suggesting that rTMS alleviated the activation of microglia and astrocytes. Another study reported that rTMS and rTMS combined with bone marrow mesenchymal stem cell transplantation could decrease GFAP expression and improve the recovery of motor function [19].

Inflammation is another inhibitor of the microenvironment in SCI. rTMS can decrease the levels of inflammatory factors, including IL-1β, interleukin-6, and TNFα, at the injured site [20]. Moreover, rTMS induced a decrease in the macrophage activation marker CD68 and an increase in IL-10 (anti-inflammatory) in the injured spinal cord. Furthermore, this study revealed that rTMS intervention for SCI could enhance brain functional connectivity and activate the HPA axis, which could induce glucocorticoid secretion and then mitigate the excessive inflammatory response. This discovery provides a potential mechanism for the analgesic effect of rTMS [22].

Additionally, deficient intrinsic neuronal growth competence also restricts recovery after SCI. The levels of proteins associated with neuronal survival, neuronal growth, axonal regeneration, and synaptic formation, such as microtubule-associated protein 2 (MAP2), nerve growth factor (NGF), brain-derived neurotrophic factor (BDNF), growth-associated protein 43 (GAP-43), and synaptic plasticity markers, such as postsynaptic densification protein 95 (PSD-95) and synaptophysin, are upregulated in the spinal cord after rTMS therapy [19,20,21]. In addition, two studies have investigated the RAF–mitogen-activated protein kinase kinase (MAP2K) signaling pathway, which may be a potential target of rTMS for SCI. Nevertheless, the effect of rTMS on the active level of MAP2K signaling remains controversial, according to these studies [12,19].

### 3.5. Other Potential Mechanisms by Which rTMS Promotes Recovery After SCI

SCI usually causes apoptosis and necrosis of neurons, destruction of the tissue structures in the spinal cord, and subsequent inflammation (Figure 2F,G). rTMS could play a role in anti-apoptosis and neuroprotection in SCI [23]. Other potential mechanisms include ferroptosis inhibition [63], increased neuron metabolism [24], changes in cerebral blood flow [64], vascular protection and neovascularization [65]. However, direct evidence in the SCI model for these mechanisms is lacking.

## 4. Clinical Research Progress on rTMS for the Treatment of SCI

TMS is a promising neurostimulation technique with the advantage of its noninvasive characteristics. Since rTMS was initially used to treat depression, it has been applied to a wide range of neurological diseases, including stroke, Parkinson’s disease, and epilepsy [4]. A growing body of evidence suggests that rTMS effectively promotes neuroplasticity, thereby facilitating the recovery of neuronal dysfunction after SCI and alleviating complications such as spasticity and neuropathic pain.

### 4.1. Motor Dysfunction

SCI typically disrupts neural circuits in the spinal cord, resulting in significant neural dysfunction, which is one of the main clinical manifestations after SCI, deprives patients of their self-care ability in daily living and profoundly affects their quality of life. Therefore, the reconstruction and activation of neural circuits, thereby promoting motor function recovery, is one of the critical therapeutic goals [66,67].

Studies employing rTMS at the primary motor cortex (M1) have demonstrated promising outcomes in improving motor function recovery in both the upper extremity [68,69,70] and lower extremity [71] of individuals with iSCI, which can be measured by the American Spinal Injury Association impairment scale (ASI), maximal voluntary contraction (MVC), upper and lower extremity motor scores (U&LEMS), 10-meter walking test (10MWT), walking index for SCI-II (WISCI-II), etc. Table 1 presents the results of clinical trials on the efficacy of rTMS for post-SCI motor dysfunction and spasticity.

The underlying mechanism of the motor recovery effects of rTMS in patients with iSCI may be related to the increase in cortical excitability and transmission of efferent neural impulses in the spinal cord [72,73]. Accordingly, several studies reported that rTMS could induce changes in electrophysiologic indices such as MEP, sEMG, and indices of corticospinal inhibition [11,70,73,74,75]. One of these studies performed neurophysiological studies in patients treated with kinesiotherapy combined with rTMS at 20–25 Hz (K + rTMS, *n* = 26) compared with kinesiotherapy only (K group, *n* = 25) [11]. After the 5-month treatment, MEPs recorded from both the abductor pollicis brevis and tibialis anterior muscles showed significant changes in the mean amplitudes but not in latencies, suggesting a slight increase in the transmission of efferent pathways from the motor cortex to the lower spinal centers. Additionally, improvements in sEMG and MEP signals were more significant in the K + rTMS group than in the K group. Another study used a novel rTMS protocol for 5 weeks, in which double pulses were separated by 100 ms (10 Hz) at a frequency of 0.1 Hz (10 s interval). The proportion of the area of inhibition in the cortex reportedly decreases, indicating that rTMS could downregulate corticospinal inhibition within the motor cortex of patients with iSCI [73].

Furthermore, numerous studies applying rTMS as an adjuvant therapy combined with various other therapies, such as gait training, lower limb resistance training, lower limb physical therapy, and body weight-supported treadmill training (BWSTT), have demonstrated further promotion of motor function recovery in individuals with SCI [68,76,77,78]. A study applied a 5-day QuadroPulse TMS, which is composed of four pulse trains with an intertrain interval of 5–6 s, combined with exercise targeting hand or leg function. The effect of the combination treatment was more pronounced than that of QuadroPulse rTMS and exercise alone [79]. However, future studies investigating the effectiveness of rTMS combined with other therapies for SCI should employ longer intervention periods and larger study populations.

### 4.2. Spasticity

Spasticity, a common symptom among patients with SCI, can exacerbate motor dysfunction [82,83]. One of the primary mechanisms underlying spasticity after SCI is the disruption of descending inhibition signals in the spinal cord, which disturb the balance between inhibitory and excitatory supraspinal inputs that regulate segmental networks [84]. rTMS may be a potential therapy on the basis of its neuromodulatory effect. Table 1 shows the results of the clinical trials of rTMS for motor dysfunction and spasticity in SCI patients.

Most studies have applied HF-rTMS at 20 Hz to the M1 in patients with SCI. Some studies have shown recovery of spasticity and motor dysfunction and improvements in gait and daily life ability [11,72,80]. For example, a study reported that HF-rTMS over the leg motor area of patients with iSCI for 15 days could relieve spasticity measured by the modified Ashworth scale (MAS) and improve motor function and gait measured by the 10MWT, cadence, step length, and TUG. These improvements were maintained for at least 2 weeks. Following sham stimulation, however, significant improvement was found only for step length and the TUG test [72].

It is hypothesized that rTMS can modify corticospinal projections by increasing motor cortical excitability and then affecting segmental spinal excitability, which is the potential mechanism of the effect on spasticity relief. Nevertheless, the results of electrophysiology studies are heterogeneous. One study applied 20 Hz HF-rTMS over the leg motor area and reported recovery from spasticity in iSCI patients, as demonstrated by the MAS, visual analog scale (VAS), and spinal cord injury spasticity evaluation tool (SCI-SET). However, there is no change in corticospinal or segmental excitability, as measured by H_max_/M_max_, the T-reflex or the withdrawal reflex [80]. In another study, treatment with 10 Hz rTMS applied to M1 increased cortical excitability in individuals with iSCI for at least 60 min but did not affect spinal excitability or alleviate spasticity [81]. Notably, the long-term effects of rTMS on motor function and spasticity might be more significant. A study in which rTMS combined with kinesiotherapy was applied for 5 months on average reported a decrease in sEMG amplitudes at rest of the tested muscles and an increase in sEMG amplitudes during maximal contraction, which suggested a decrease in spasticity and an improvement in muscle motor unit activity [11].

### 4.3. Neuropathic Pain

Neuropathic pain is a common complication after SCI that affects approximately 40% to 50% of patients with SCI [85,86,87]. It usually develops within the first year and tends to become chronic, causing lifelong problems [88,89]. Since, in patients with SCI, the response to existing treatments for neuropathic pain is inadequate and unsatisfactory [90,91], effective pain management remains an unmet need, prompting increased focus on rTMS in this field. Table 2 shows the results of the clinical trials on the use of rTMS for the treatment of neuropathic pain after SCI.

A study conducted by Jetté et al. revealed that active rTMS applied over the motor cortex might reduce neuropathic pain after iSCI, regardless of any changes in cortical excitability of the specific region of the M1 region, suggesting that the analgesic effect might be mediated by distant mechanisms and not associated with local changes at the motor cortex level itself [92]. Another study, however, demonstrated the analgesia-enhancing effects of HF-rTMS over M1. The suppression of the M1 and premotor cortex (PMC) after HF-rTMS therapy measured with functional near-infrared spectroscopy (fNIRS) indicated that the analgesia-enhancing effects might be associated with the amelioration of M1 and PMC hypersensitivity [93]. A previous study revealed the analgesic effect of rTMS, which targets the premotor cortex/dorsolateral prefrontal cortex (PMC/DLPFC) in patients with SCI [94].

Furthermore, the analgesic effect of rTMS may also involve changes in neurotrophic factor levels. Zhao et al. applied rTMS at the hand area of the motor cortex in patients with acute neuropathic pain after SCI. This treatment alleviated acute neuropathic pain, as measured by a numeric rating scale (NRS) and the short-form McGill pain questionnaire-2 (SF-MPQ2-CN). Along with the analgesic effect, serum BDNF and NGF levels increase [95]. Both BDNF and NGF are crucial for neural maintenance and repair; however, they also contribute to the sensitization of pain pathways.

Despite these promising findings, there remains controversy surrounding the analgesic effect of rTMS in patients with SCI [96,97,98,99,100]. Some studies reported that while the pain-relief effect of rTMS was not significantly different from that of sham stimulation, rTMS could have antinociceptive effects, anxiolytic effects, and antidepressant effects [101,102]. These results showed that rTMS could modulate the different dimensions of pain in patients with SCI without necessarily affecting clinical pain per se. Therefore, further research is warranted to comprehensively evaluate the effectiveness and mechanisms of rTMS for the management of neuropathic pain in SCI patients.

**Table 2:** Clinical studies on rTMS for the treatment of neuropathic pain after SCI.

Title	(Authors, Year)	Participants				Blinding	Region of Stimulation	TMS Protocol			Outcome Timing	Main Outcome Measures	Conclusion
		Sample Size	Level of SCI	ASI	Course of SCI			Frequency	Intensity	Pulses, Treatment Cycle			
Effect of single-session repetitive transcranial magnetic stimulation applied over the hand versus leg motor area on pain after spinal cord injury	[92]	16	C1-T12	A–D	more than 2 years after SCI	DB	hand primary motor cortex leg primary motor cortex	10 Hz	90% of the FDI RMT over the hand area 110% of the FDI RMT over the leg area	3 single sessions, a total of 2000 stimuli	0 and 20 min past rTMS a week before and a week after each session	NRS, parameters derived from the motor mapping of the first dorsal interosseous muscle (including maximal amplitude of evoked response as well as map area, volume, and location)	rTMS applied over the hand or leg motor cortex decreased neuropathic pain regardless of any change in cortical excitability, suggesting that the analgesic effect is not associated with local changes at the motor cortex level itself.
Analgesia-enhancing effects of repetitive transcranial magnetic stimulation on neuropathic pain after spinal cord injury: An fNIRS study	[93]	17	C3-L1	A–D		DB	left primary motor cortex corresponding to the hand area	10 Hz	80% RMT	1200, six weeks with a one-day interval per week	baseline after the first session of rTMS (day 1) at the end of week 1, 2, 4, and 6	NRS, fNIRS	The analgesia-enhancing effects of HF-rTMS might be related to the amelioration of M1 and PMC hypersensitivity in patients with SCI.
rTMS of the prefrontal cortex has analgesic effects on neuropathic pain in subjects with spinal cord injury	[94]	12	C5-T10	A–D	more than 4 years after SCI	DB	PMC/DLPFC	10 Hz	120% RMT	1250, 5 days per week for 2 weeks.	baseline 1 day after the first week of treatment, 1 day, 1 week and 1 month after the last intervention	VAS, MPQ, HAM-D, HAM-A	rTMS of the PMC/DLPFC may be effective in relieving neuropathic pain in SCI patients.
Analgesic Effects of Directed Repetitive Transcranial Magnetic Stimulation in Acute Neuropathic Pain After Spinal Cord Injury	[95]	48	below C8		less than 3 months before	DB	hand area of the motor cortex	10 Hz	90% RMT	1500, 6 days per week, for a total of 3 weeks	baseline three days (T1), one week, two weeks, and three weeks after onset of treatment	NRS, SF-MPQ2-CN, BDNF, NGF, MEP, maximal amplitude	Ten-Hz rTMS over the hand area of the motor cortex could alleviate acute CNP in the early phase of SCI, enhance MEP parameters and modulate BDNF and NGF secretion.
Neurogenic pain relief by repetitive transcranial magnetic cortical stimulation depends on the origin and the site of pain	[98]	60 (12 with SCI)				SB	motor cortex corresponding to the hand on the painful side	10 Hz	80% RMT	1000 (single session)	before and after the session of rTMS	thermal sensory thresholds, VAS	Motor cortex rTMS was found to result in a significant but transient relief of chronic pain influenced by pain origin and pain site.
Effect of repetitive transcranial magnetic stimulation over the hand motor cortical area on central pain after spinal cord injury	[99]	11	C6-T11	A–D	more than 15 months after SCI	DB	hand area of the motor cortex	10 Hz	80% RMT	1000, 5 days	baseline immediately after the third and fifth stimulation sessions 1, 3, 5, and 7 weeks after the end of the 5-day stimulation period	NRS, BPI	The therapeutic efficacy of rTMS was not demonstrated when rTMS was applied to the hand motor cortical area in patients with chronic neuropathic pain at multiple sites in the body.
The effect of repetitive transcranial magnetic stimulation on refractory neuropathic pain in spinal cord injury	[100]	17			more than a year	DB	The vertex (projection of motor cortex area corresponding to the lower extremities)	10 Hz	110% RMT	1500,	baseline 10 days, 6 weeks and 6 months after the treatment	VAS, five-point Likert scale	Our results demonstrated that the analgesic effect of rTMS on intractable neuropathic pain in SCI was not superior to placebo. However, middle-term (over 6 weeks) pain relief by rTMS is encouraging and suggests the need for future studies with a larger sample size.
The Effect of a Series of Repetitive Transcranial Magnetic Stimulations of the Motor Cortex on Central Pain After Spinal Cord Injury	[101]	11	T4-T12			DB	motor cortex in which the legs and lower back are represented	5 Hz	115% motor session	500, 10 days	baseline after the 10 daily sessions of rTMS 2–6 weeks after the last rTMS session	VAS, MPQ, pain threshold, BDI	Although the acute effect of rTMS observed herein is probably due to a placebo effect, the long-term effects may have true clinical significance for pain relief in SCI patients.
Insular and anterior cingulate cortex deep stimulation for central neuropathic pain: Disassembling the percept of pain	[102]	98 (after stroke or SCI)				DB	ACC, PSI	10 Hz	90% RMT	1500, induction sessions: 5 consecutive days maintenance sessions: weekly until the 12th week (total of 16 sessions)	baseline after the end of the first, second, and third months of treatment	NRS	ACC- and PSI-dTMS were not different from sham-dTMS for pain relief in CNP despite a significant antinociceptive effect after insular stimulation and anxiolytic effects of ACC-dTMS.

PMC, premotor cortex; DLPFC, dorsolateral prefrontal cortex; ACC, anterior cingulate cortex; PSI, posterior superior insula; NRS, numeric rating scale; fNIRS, functional near-infrared spectroscopy; MPQ, McGill pain questionnaire; HAM-D, Hamilton rating scale for depression; HAM-A, Hamilton anxiety rating scale; SF-MPQ2-CN, short-form McGill pain questionnaire-2 (Chinese Edition); BDNF, brain-derived neurotrophic factor; NGF, nerve growth factor; BPI, brief pain inventory; BDI, Beck depression inventory.

## 5. Novel Protocols Involving TMS for the Treatment of SCI

The variety of TMS parameters can lead to different intensities and polarities of modulation, influencing neurophysiology and clinical effects. On the basis of the critical parameters of geometry and timing [37], TMS has generated a variety of novel protocols applied for SCI treatment, including iTBS and PAS.

### 5.1. iTBS

**Figure 3:**
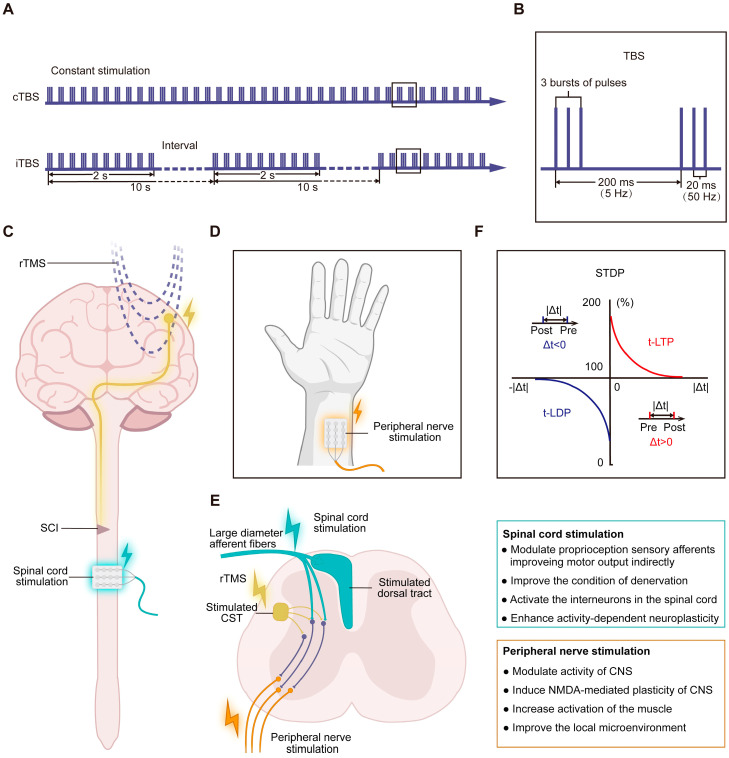
Novel protocols of TMS for the treatment of SCI: (**A**) TBS is composed of three bursts of pulses at 50 Hz every 200 ms (5 Hz), which can be classified into continuous TBS (cTBS) and intermittent TBS (iTBS), which induce LTD-like effects and LTP-like effects, respectively. LTD, long-term depression; LTP, long-term potentiation. The boxed areas in (**A**) are magnified in (**B**). (**B**) TBS pattern. (**C**) Paired associative stimulation (PAS) combined with rTMS and spinal cord stimulation. (**D**) PAS combined with rTMS and peripheral nerve stimulation. (**E**) Neuromodulation effects of the PAS on circuits and functional connections in the spinal cord. rTMS can modulate functional connections between the brain and spinal cord, activate the cortex and CST and recruit circuits in the spinal cord. Spinal cord stimulation (SCS) mainly depolarizes large-diameter afferent fibers, especially in the dorsal root entry zones, modulating proprioception sensory afferents, which can enhance the connection of circuits in the spinal cord and improve motor output indirectly (top box on the right). Peripheral nerve stimulation (PNS) may also change the activity of the CNS and produce local effects on muscle function and the peripheral microenvironment (lower box on the right). PAS may be based on the synergistic effect of rTMS and SCS or PNS. (**F**) Schematic diagram presenting classic spike-timing-dependent plasticity (STDP). *X*-axis, the interval between the PNS and TMS (Δt); *Y*-axis, the relative magnitude of change in the synaptic weight, is an indicator of synaptic plasticity. When presynaptic action potentials (pre) arrive prior to postsynaptic depolarizing action potentials (post) (Δt > 0), synaptic plasticity is enhanced, which is known as timing-dependent LTP (t-LTP); conversely, if pre arrive after post (Δt < 0), synaptic plasticity is decreased, which is known as timing-dependent long-term depression (t-LTD).

TBS is a special protocol based on endogenous oscillations at the theta frequency in the hippocampus involving learning, memory and movement [37]. It is composed of three bursts of pulses at 50 Hz every 200 ms (5 Hz). On the basis of these patterns, TBS can be classified into continuous TBS (cTBS) and iTBS, which induce LTD-like (inhibitory) effects and LTP-like effects (facilitatory), respectively (Figure 3A,B). With this facilitatory effect, iTBS has become one of the potential and innovative protocols of TMS currently under study (Table 3).

Amer et al. reported that a single iTBS block and concatenating five iTBS blocks at the motor cortex could produce MEP LTP lasting ~30–45 min and 24–48 h, respectively, in normal rats (without SCI), which might be dependent on CamKIIα. When the rats were treated with daily iTBS for 10 days, however, the MEP enhancement lasted for at least 10 days, paralleling CST axonal outgrowth and indicating potential sites of synapses and structural changes at the CST-spinal interneuron synapses, such as increases in the number and volume of postsynaptic clusters [103].

Another preclinical study with SCI rats also demonstrated that iTBS for 2 weeks could increase the levels of MEP and GAP-43, indicating synaptic plasticity, axonal regeneration, and synaptic formation. However, locomotor function, as measured by the Basso Beattie Bresnahan scale (BBB), was not significantly better in the rats treated with iTBS than in the rats in the sham-iTBS group.

There are several clinical studies on the use of iTBS for SCI [25,26,104]. A clinical trial reported that iTBS could increase biceps corticomotor excitability in individuals with tetraplegia, as predicted by the corticomotor conductance potential [25]. Yang et al. compared the analgesic effects of iTBS, 10 Hz rTMS, and iTBS + rTMS. The results demonstrated that both iTBS and rTMS could relieve pain, with no statistically significant differences between them. However, iTBS requires less time than rTMS does, reducing the burden on patients. Moreover, iTBS as a priming stimulus was found to be more effective than iTBS as a standalone procedure [104]. Some studies have also combined iTBS with precise root stimulation [105] or trans-spinal direct current stimulation (tsDCS) [106].

**Table 3:** Clinical studies on novel protocols of TMS for the treatment of SCI.

Title	(Authors, Year)	Participants				Blinding	Novel Protocol		Outcome Timing	Main Outcome Measures	Conclusion
		Sample Size	Level of SCI	ASI	Course of SCI		Pattern	Treatment Cycle			
**iTBS**											
Intermittent theta burst stimulation modulates biceps brachii corticomotor excitability in individuals with tetraplegia	[25] ^#^	10	C5–C8	A–D	more than 2 years after SCI	SB	iTBS (motor areas)	3 independent sessions	baseline 10, 20 and 30 min after iTBS	MEP	Preliminary results suggest that iTBS increases biceps corticomotor excitability in individuals with tetraplegia with effects that may be predicted by corticomotor conductance potential.
Cerebral Theta-Burst Stimulation Combined with Physiotherapy in Patients with Incomplete Spinal Cord Injury: A Pilot Randomized Controlled Trial	[26]	38		C–D	approximately 3 months on average after SCI	DB	iTBS	5 times per week, 9 weeks	baseline 9 weeks after the start of the intervention	LEMS, RMS, RMS of the quadriceps femoris muscle, walking speed, stride length, HWAS, MBI,	Cerebral intermittent theta-burst stimulation with physiotherapy promotes lower extremity motor recovery in patients with incomplete spinal cord injury.
Effects of different transcranial magnetic stimulations on neuropathic pain after spinal cord injury	[104]	37		B–D	approximately 15 months on average after SCI	DB	iTBS, rTMS, iTBS + rTMS (M1)	5 days a week, 4 weeks	1 day before stimulation (baseline) 1 day after the first week of stimulation 1 day after the last stimulation	VAS, HAM-D, PSQI	The three different modalities were all effective at relieving the pain. There were statistical differences in the treatment of neuropathic pain between iTBS as a priming stimulus and as a single procedure.
Effects of cortical intermittent theta burst stimulation combined with precise root stimulation on motor function after spinal cord injury: a case series study	[105]	14	C3-L1	C–D	more than 6 months, less than 10 years after SCI		iTBS (right M1) + precise root magnetic stimulation (bilateral L3-L4)	5 times a week, 4 weeks	baseline 4 weeks of treatment	rMEP, CMCT, LEMS, Berg balance score, SCIM-III 10 m-walking speed	Cortical iTBS combined with precise root stimulation can improve nerve conduction of the corticospinal tract and lower limb motor function recovery in patients with chronic SCI.
Effectiveness of Repetitive Transcranial Magnetic Stimulation Combined With Transspinal Electrical Stimulation on Corticospinal Excitability for Individuals With Incomplete Spinal Cord Injury: A Pilot Study	[106]	12	C4-T6	A–D	more than 1 year after SCI	DB	tsDCS + iTBS, iTBS, tsDCS	8 weeks	baseline 4, 8, and 12 weeks since the beginning of the intervention	MEP, LEMS, RPM, RMS	The combination of iTBS and tsDCS treatment was more effective than iTBS alone or tsDCS alone in enhancing corticospinal excitability.
**PAS**											
Motor Recovery after Spinal Cord Injury Enhanced by Strengthening Corticospinal Synaptic Transmission	[27]	19	C4–C8		more than 1 year after SCI		TMS + PNS	100 pairs	before and after (0, 10, 20, and 30 min) the paired-pulse stimulation protocols	F-wave, MEP, raw force and EMG traces, 9HPT	The arrival of corticospinal volleys prior to motoneuron discharge at residual corticospinal-motoneuronal synapses will enhance voluntary motor output in humans with chronic iSCI.
Potentiating paired corticospinal-motoneuronal plasticity after spinal cord injury	[28]	17	C3–C8	A–D	more than 1 year after SCI		TMS + PNS (PCMS_rest_ and PCMS_active_)	100 pairs	before, immediately after (0 min) and 30 min after PCMS_rest_ and PCMS_active_	MEP	Muscle contraction during PCMS potentiates corticospinal transmission. PCMS applied during voluntary activity may represent a strategy to boost spinal plasticity after SCI.
Increased paired stimuli enhance corticospinal-motoneuronal plasticity in humans with spinal cord injury	[107]						TMS + PNS (PCMS)	180 pairs and 360 pairs	before and after PCMS paired pulses	MEP, 9HPT	Increasing the number of PCMS-paired pulses potentiates corticospinal excitability and voluntary motor output after SCI, likely through spinal plasticity.
Corticospinal-motor neuronal plasticity promotes exercise-mediated recovery in humans with spinal cord injury	[108] ^#^	38	C2-L3	A–D	more than 1 year after SCI		TMS + PNS (PCMS)	10 sessions of treatment completed in 2–3 weeks	before and after 10 sessions 6 months after the treatment	MEP, MVC, CCT, PCT, GRASSP, 10MWT,	PCMS represents a strategy to boost residual corticospinal connections and preserve exercise-mediated recovery in humans with different degrees of paralysis and levels of SCI.
Effects of Long-Term Paired Associative Stimulation on Strength of Leg Muscles and Walking in Chronic Tetraplegia: A Proof-of-Concept Pilot Study	[109]	5	C1–C5	D	more than 2 years after SCI	DB	TMS + PNS (PAS)	5 times per week during the first 2 weeks and 3 times per week for the subsequent 6 months	baseline after 1 and 2 months of PAS after a 1-month follow-up	MMT, AIS, MAS, SCIM	The results of this proof-of-concept pilot study justify a larger trial on the effect of long-term PAS on leg muscle strength and walking in people with chronic incomplete SCI.
Brain and spinal cord paired stimulation coupled with locomotor training affects polysynaptic flexion reflex circuits in human spinal cord injury	[110] ^#^	8	C4-T12	A–D	more than 1 year after SCI	SB	TMS-trans-spinal PAS, trans-spinal-TMS PAS during assisted step training	1 h/day, 5 days/week, ~5 weeks	before and after PAS and locomotor training	EMG traces of TA, TA flexion reflex, treadmill and robotic-gait orthosis settings	Transspinal and TMS PAS warrant further investigation to understand better sites of neural actions that may lead to the development of more suitable protocols that can effectively maximize the activity-dependent neuroplasticity produced by exercise and stimulation in people with SCI.
Brain and spinal cord paired stimulation coupled with locomotor training facilitates motor output in human spinal cord injury	[111] ^#^	10	C2-T11	A–D		SB	TMS-trans-spinal PAS, trans-spinal-TMS PAS during assisted step training	1 h/day, 5 days/week, ~5 weeks	before and after PAS and locomotor training	TEP, training intervention parameters.	Activity-based brain and spinal cord stimulation can increase the net motor output, improving motor ability in people with chronic SCI.
Neurophysiological Changes After Paired Brain and Spinal Cord Stimulation Coupled With Locomotor Training in Human Spinal Cord Injury	[112] ^#^	10	C4-T12	A–D	more than 1 year after SCI	SB	TMS-transcutaneous spinal cord, stimulation (trans-spinal) PAS, trans-spinal-TMS PAS during assisted step training	1 h/day, 5 days/week, ~5 weeks	before and after PAS and locomotor training	EMG, H-reflex, M-waves, H_max_/M_max_, training intervention parameters	Noninvasive trans-spinal and TMS PAS warrant further investigation as an intervention that potentially may lead to improvements in leg function and supplement the benefits of locomotor training in people with SCI.

iTBS, intermittent theta burst stimulation; tsDCS, trans-spinal direct current stimulation; PAS, paired associative stimulation; PNS, peripheral nerve stimulation; PCMS, paired corticospinal-motor neuronal stimulation; RMS, root–mean square; HWAS, Holden walking ability scale; MBI, modified Barthel index; PSQI, Pittsburgh sleep quality index; rMEP, resting MEP; CMCT, central motor conduction time; SCIM-III, spinal cord independence measure-III scale; RPM, revolutions per minute; 9HPT, nine-hole peg test; CCT, central conduction time; PCT, peripheral conduction time; GRASSPE, graded and redefined assessment of strength, sensibility and prehension; ^#^ Study combined rTMS with conventional rehabilitation therapy such as kinesiotherapy.

### 5.2. PAS

Another novel protocol, the PAS, combines TMS with PNS, SCS, or precise root stimulation (Figure 3C–E) and is supposed to enhance the efficacy of a single pattern of stimulus (Table 3).

PNS has been demonstrated to have multiple benefits, including modulating the activity of the CNS [113,114,115], increasing the levels of neurotransmitters [116], inducing NMDA-mediated plasticity in the CNS [114], and improving muscle function [117] and the local microenvironment [118,119] (Figure 3D,E). The paired-pulse stimulation composed of TMS and the PNS is the traditional PAS pattern (Figure 3D,E), which is based on spike-timing-dependent plasticity (STDP), a process of precisely timing the arrival of presynaptic action potentials prior to postsynaptic depolarizing action potentials (Figure 3F). It is also known as paired corticospinal–motor neuronal stimulation (PCMS). It has been reported that PCMS can potentiate synaptic strength and motor output. Several studies have reported that PCMS could enhance MEPs and potentiate corticospinal transmission according to electrophysiological techniques [27,28]. A study demonstrated that increasing the number of PCMS-paired pulses could improve the effects of stimulation on corticospinal excitability and voluntary motor output after SCI, which likely depends on spinal plasticity [107]. Jo et al. compared the treatment of PCMS + exercise with PCMS and exercise alone for SIC. All three therapies could decrease the time to complete subcomponents of the graded and redefined assessment of strength, sensitivity, and prehension (GRASSP) and the 10MWT average by 20%, and only the PCMS with or without exercise could significantly increase the MVC. Moreover, PCMS can preserve exercise-mediated recovery after SCI [108]. However, larger trials on the effects of PCMS in patients with SCI are needed [109].

Precise nerve root stimulation is another novel stimulation technique. It has been reported that nerve root magnetic stimulation can induce locomotor function recovery, electrophysiological improvements, and cortical synaptic reconstruction in an SCI rat model [120]. Mao et al. combined cortical iTBS with precise root stimulation. This therapy for 4 weeks improved corticospinal excitability and motor function recovery in patients with SCI [105].

It has been reported that the SCS can depolarize large-diameter afferent fibers, especially in the dorsal root entry zones, which can recruit and activate motor neurons and spinal circuits, induce activity-dependent regeneration, and promote functional recovery [7] (Figure 3C,D). There are several types of SCS, including epidural spinal stimulation, intraspinal stimulation (experimental), and tSCS. tSCS, as a noninvasive stimulation strategy, has garnered increasing interest. Compared with the PNS, tSCS can increase corticospinal excitability, influencing functional motor output in healthy participants [121]. Pulverenti et al. combined rTMS + tSCS with robotic-assisted step training and reported that this therapy could induce a change in the trans-spinal evoked potential (TEP), facilitate motor output, and affect the polysynaptic flexion reflex [110,111,112]. Animal experiments and clinical trials have applied cortical iTBS + tsDCS and rTMS + ts-iTBS to SCI, which significantly improved corticospinal excitability and locomotion outcomes, such as the MEP and LEMS.

## 6. Perspectives

rTMS, a noninvasive brain stimulation technique, is considered a potential treatment for SCI [6,7,8]. Mounting evidence suggests that rTMS can persistently increase corticospinal output [13,15] via spared descending circuits, facilitate neural regeneration, and induce reorganization of corticospinal circuits and interneuron circuits [11,12,27]. Additionally, rTMS has the potential to alleviate complications associated with SCI, such as spasticity [80] and neuropathic pain [92,93]. However, studies on the underlying mechanism of rTMS therapy for SCI are still not sufficient or in-depth. There is a need for more comprehensive investigations at multiple levels, including investigations of the cortex and neural circuits, cellular processes, genomics and molecular mechanisms.

Notably, despite advancements, the safety and efficacy of rTMS applied to SCI patients remain controversial because of the high degree of heterogeneity among the studies, which is one of the barriers to the clinical application of rTMS to SCI patients. The small sample size of these studies is one of the critical factors. Therefore, large-sample multicenter randomized controlled trials are necessary to establish the safety and efficacy of rTMS conclusively. It is important to note that although rTMS is widely recognized as a relatively safe technique, a small proportion of individuals may still experience adverse reactions such as headaches, hearing issues, and seizures. However, most of the side effects are short-term in nature, and reports of long-term side effects related to rTMS are rare. rTMS also has certain contraindications, including the presence of metallic implants, a history of seizures, and mental health disorders [122,123].

Economic and time costs also influence the promotion of rTMS [123,124]. However, large-scale, long-term studies assessing the general economic impact of rTMS on SCI rehabilitation are lacking. The cost-effectiveness of rTMS may vary significantly based on factors such as the severity of SCI, treatment prescriptions, the effects of integrative therapy, insurance coverage, the accessibility of rTMS equipment and trained professionals, etc. Although rTMS may increase the economic burden in the short term, it has the potential to shorten the course of traditional drug treatment and physical therapy. Furthermore, it may reduce healthcare costs in the long term by promoting recovery and improving prognosis [67]. Nevertheless, further research is required to comprehensively evaluate the long-term cost-effectiveness of rTMS in SCI rehabilitation, particularly in comparison to conventional therapies.

Additionally, in current studies, there are significant inconsistencies in the protocols of rTMS, including variations in parameters, stimulus sites, and treatment courses. The methods used to establish animal models and participant conditions in clinical studies are also highly variable. Furthermore, the diverse selection of measures employed to evaluate outcomes impedes the feasibility of synthesizing and conducting comparative analyses of the results across different studies. Hence, to understand the mechanism and clinical application of rTMS for SCI, relatively optimized and standardized protocols and efficacy assessment methods are imperative. In recent years, numerous studies have focused on optimizing the protocols of rTMS, as well as exploring innovative protocols such as iTBS [25,26,105] and PAS [28,108,111], which may promote further development of rTMS. Indeed, the optimization of the protocol strongly depends on the progress of mechanism research. For example, coil geometry, precise location, and pulse characteristics may be associated with specific circuits and neuronal populations; stimulus frequency timing and amplitude may be related to the electrophysiological properties of the targeted neurons [37].

Furthermore, repetitive magnetic stimulation techniques can also be applied to peripheral nerves. Repetitive peripheral magnetic stimulation (rPMS) cannot deliver a controlled focal stimulus like conventional electrical stimulation; however, the magnetic field can reach deeper neural structures, and its stimulation range is usually wider, allowing it to affect a larger group of nerves [125,126]. There have been several clinical studies that applied rPMS to people after stroke [125,127,128], and it was reported that rPMS applied over the upper and lower limbs could improve limb function and alleviate spasticity in individuals post-stroke. Another clinical study applied rPMS over the lumbar area of patients who have lumbar radiculopathy, with or without kinesiotherapy. The result showed a reduction in pain in both groups and superior functional recovery in the rPMS + kinesiotherapy group [129]. These findings suggest that rPMS can be an alternative noninvasive PNS or SCS technique paired with rTMS to improve the effect of neuromodulation. Although there is no research supporting the role of paired associative magnetic stimulation (PAMS) in SCI treatment, an existing study has demonstrated that PAMS can increase regional brain activity, enhance neuroplasticity, and promote functional recovery in the rat middle cerebral artery occlusion (MCAO) model [130]. In addition to the respective effects of rTMS and rPMS, they may also exhibit synergistic mechanisms and effects. According to the existing research, PAMS is believed to adhere to the principles of STDP [131] and induce LTD [130], which plays a crucial role in synaptic plasticity. Additionally, PAMS may establish a connection for neuromodulation at the cortical level, neural circuit level, and local spinal or peripheral nerve level [130,132].

Considering that the effect of a single therapy for SCI is usually not sufficient, combination therapy could be a potential development direction for the treatment and rehabilitation of SCI [2]. Thus, rTMS can be applied as an adjuvant therapy alongside other interventions, such as rehabilitation, movement therapy, and other modality therapy, as well as biological strategies, such as bioactive substance regulation, biomaterial implantation, and cell therapy.

Among the alternative combination therapies involving rTMS for SCI, NPCs represent a potentially powerful option. Accompanied by advancements in cellular engineering, NPCs are available from a variety of tissues and cells (fetal or adult CNS tissues, embryonic stem cells, induced pluripotent stem cells, etc.) [133] and have been verified to survive long-term at the SCI site [31]. Furthermore, with substantial progress in understanding the pathological process involved in SCI, neuroplasticity and circuit reorganization, the promotion of nerve regeneration and the formation of relay pathways to restore function have become the primary goals for restoring neurological functions [53,133,134]. According to the existing mechanisms, NPCs and rTMS may have synergistic effects on this process.

On the one hand, both NPCs and rTMS effectively promoted regeneration of the host axons. NPC grafts can prolong the embryonic transcriptional growth state induced by SCI and promote the regeneration of host axons [29,30]. rTMS has also been shown to enhance the intrinsic regeneration ability of host neurons [12]. On the other hand, graft-derived neurons can integrate into the injured site both structurally and functionally. The regeneration of host axons into grafts and the formation of host-to-graft and graft-to-host synapses are observed after transplantation [30,31,32], which compensates for insufficient host-axon regeneration to reconstruct circuits. Moreover, graft-derived glial cells can support neurons from both the graft and the host, alleviate inflammation [135,136], reduce scar formation [137], and promote survival, regeneration and remyelination. rTMS may also improve the microenvironment and protect neurons in the spinal cord [20,22]. These synergistic effects provide structural foundations and conditions for circuit reorganization.

At the level of functional connectivity, rTMS provides neuromodulation, which is necessary for connectivity reinforcement and circuit reconstruction involving host neurons and NPC grafts. As circuits are disrupted by SCI, the functional map and activity from the cortex to guide circuit reconstruction are lost or changed [7]. In fact, robust evidence suggests that the process of rehabilitation after SCI largely depends on increased exogenous modulation or volitional control to restore functional connections [10,53,138]. Thus, rTMS, as a technique of neuromodulation, has the potential to induce changes in the cortical map and increase the activity-dependent neuroplasticity of the host axons and NPC grafts, improving connectivity and function. Additionally, losing maps and afferents can lead to spontaneous maladaptive reorganization of the host axons, propriospinal neurons, and NPC grafts, especially below the injured site [7]. The maladaptive reorganization cannot restore functions; however, it can induce various clinical syndromes, including spasticity, neuropathic pain, and autonomic dysfunction. rTMS may provide afferents to the SCI site, and the NPC graft may relay the pathway to the distal deafferent region, reducing maladaptive reorganization, which could be a possible mechanism underlying the alleviation of spasticity and neuropathic pain. Although several mechanisms support the combination of rTMS and NPC transplantation, more remains to be revealed. Furthermore, practical protocols and clinical effects have not been studied in depth.

## Figures and Tables

**Table 1:** Clinical studies on rTMS for the treatment of motor dysfunction and spasticity after SCI.

Title	(Authors, Year)	Participants				Blinding	Region of Stimulation	TMS Protocol			Outcome Timing	Main Outcome Measures	Conclusion
		Sample Size	Level of SCI	ASI	Course of SCI			Frequency	Intensity	Pulses, Treatment Cycle			
The Long-Term Effect of Treatment Using the Transcranial Magnetic Stimulation rTMS in Patients after Incomplete Cervical or Thoracic Spinal Cord Injury	[11] ^#^	55	C2-T12	C–D			bilateral motor cortex	20–25 Hz	70–80% RMT	1600 (biphasic), 3–5 sessions in a month, 5 months on average	baseline 2 to 3 months after rTMS 5 months after rTMS	sEMG and MEP	rTMS can promote recovery of motor control in iSCI patients.
Placebo-controlled study of rTMS combined with Lokomat^®^ gait training for treatment in subjects with motor incomplete spinal cord injury	[68] ^#^	31	C3-T11	C–D	from 2 weeks to 6 months after SCI	DB	over the vertex	20 Hz (40 pulses/burst)	90% RMT	1800, one daily session for 20 days	baseline after the last rTMS session (4 weeks) during follow-up (8 weeks)	MAS, U&LEMS,10MWT, WISCI II	Twenty sessions of daily HF-rTMS combined with Lokomat gait training can lead to clinical improvement of gait and motor strength in the lower extremity in iSCI subjects and in the upper extremity in those with cervical SCI.
Improvements in hand function in adults with chronic tetraplegia following a multiday 10-Hz repetitive transcranial magnetic stimulation intervention combined with repetitive task practice	[69] ^#^	11	C7 or above			DB	primary motor cortex	10 Hz	80% RMT	800, 3 days	before and after treatment	JTT, pinch and grasp strength, MEP	rTMS may be a valuable adjunct to RTP for improving hand function in persons with tetraplegia. Higher stimulation doses (frequency, intensity, number of sessions) may be associated with larger effects.
Treatment of patients with cervical and upper thoracic incomplete spinal cord injury using repetitive transcranial magnetic stimulation	[70]	15	C4-T2	B–D	from 5 months to less than a year after SCI		bilateral primary motor cortex	20–22 Hz	70–80% RMT	1600 (biphasic), 3–5 sessions per month, 5–6 months	before and after each treatment section	sEMG, MEP	rTMS reduced the increased muscle tension in upper extremity muscles, improved the function of upper extremity muscle motor units and slightly improved the transmission of efferent neural impulses within spinal pathways. rTMS may also inhibit inevitable pathological changes in nerves.
The effect of high-frequency repetitive transcranial magnetic stimulation on motor recovery and gait parameters in chronic incomplete spinal cord injury: A randomized-controlled study	[71]	28		C–D	more than 1 year after SCI	DB	primary motor cortex	20 Hz	110% RMT	1600,	baseline, three weeks (post-treatment) and five weeks (follow-up) after the treatment.	LEMs, three-dimensional gait analysis, WISCI II, 10MWT	The study findings support the therapeutic effectiveness of rTMS on motor recovery in chronic iSCI.
Motor and Gait Improvement in Patients With Incomplete Spinal Cord Injury Induced by High-Frequency Repetitive Transcranial Magnetic Stimulation	[72]	17	C4-T12	D	3–12 months after SCI	DB	leg primary motor cortex	20 Hz	90% RMT	800, 15 days	baseline after the last sessions 2 weeks after the last rTMS session	LEMS, MAS, 10MWT, WISCI II, step length, cadence, TUG	HF-rTMS over the leg motor area can improve LEMS, spasticity, and gait in patients with motor iSCI.
Magnetic brain stimulation can improve clinical outcome in incomplete spinal cord injured patients	[73]	4	C5	D	duration of injury 15 months, or 7–8 years	DB	left motor cortex	0.1 Hz	90% threshold for evoking MEP	360 doublet pulses separated by 100 ms (10 Hz), 5 days a week, 3 weeks	baseline period 2 sessions (sham and rTMS) sham treatment week 2 sessions therapeutic treatment week 4 sessions follow-up period: weekly for 3 weeks	index of corticospinal inhibition, ASI, perceptual threshold, timed nine-hole peg board	rTMS has been shown to alter cortical inhibition in iSCI and improve clinical and functional outcomes.
Action of 5 Hz repetitive transcranial magnetic stimulation on sensory, motor and autonomic function in human spinal cord injury	[74]	15	C2–C8	A–D	more than 3 years after SCI	SB	motor cortex	5 Hz	80% AMT	900, 5 days	before and 2 weeks after treatment	ASI, ARAT, peg-board test, EPT, MEP, cortical silent period, cardiovascular, sympathetic skin responses	Changes in cortical motor threshold measures may accompany functional gains to rTMS in SCI subjects.
Effects of Repetitive Transcranial Magnetic Stimulation on Motor Recovery in Lower Extremities of Subacute Stage Incomplete Spinal Cord Injury Patients: A Randomized Controlled Trial	[75]	19	C4-T9	C–D	no more than 6 months since SCI	DB	motor cortex of the lesional hemisphere	10 Hz	80% AMT	5 days per week, 6 weeks	before and after the therapy period	latency, amplitude, MNCV	rTMS may be beneficial in improving motor recovery in the lower extremities of subacute stage SCI patients.
Therapeutic Application of Transcranial Magnetic Stimulation Combined with Rehabilitative Training for Incomplete Spinal Cord Injury: A Case Report	[76] ^#^	1	C6	D	76 days after SCI		leg primary motor cortex	10 Hz	110% RMT	1500,30 sessions over 19 days.	before and after treatment	ASI, knee extensor muscle strength, maximum calf circumference of bilateral lower legs, MAS, ADL, SPPB, ABMS-2, FIM	This case study demonstrated the safety and feasibility of TMS combined with rehabilitative training in a patient with iSCI.
Effects of repetitive transcranial magnetic stimulation on recovery in lower limb muscle strength and gait function following spinal cord injury: a randomized controlled trial	[77] ^#^	20	C2-L2	A–D		DB	leg primary motor cortex	20 Hz	100% RMT	1800, 5 days a week, 4 weeks	baseline the day after the last session LEMS was performed at admission and within 1 week of discharge	MVC, LEMS, 10MWT, TUG, 6MWT	HF-rTMS may increase long-term training-induced recovery of lower limb muscle strength following SCI. The effect on short-term recovery is unclear. Four weeks of rTMS, when delivered in conjunction with resistance training, has no effect on recovery of gait function, indicating a task-specific training effect.
Independent community walking after a short protocol of repetitive transcranial magnetic stimulation associated with body weight-support treadmill training in a patient with chronic spinal cord injury: a case report	[78] ^#^	1	T8	D	8.5 years after traumatic iSCI		over the vertex	10 Hz	90% RMT	1800, 3 days a week, 4 weeks	before and after 12 sessions of rTMS	WISCI II, LEMS, SF-36, MAS	A short protocol of rTMS combined with BWSTT improved walking independence, motor function, spasticity, functional mobility and quality of life in this patient with iSCI.
Efficacy of QuadroPulse rTMS for improving motor function after spinal cord injury: Three case studies	[79] ^#^	3	C5–C7	B–D	more than 9 months after SCI		the hand area of the contralateral motor cortex	250 Hz, 500 Hz	120% RMT	250–360 trains, 5 days	before and after treatment	PPT, CMT, TM, TUG, MEP, T-response size	There is a functional benefit of motor cortical QuadroPulse rTMS, which appears to be augmented when stimulation is accompanied by targeted exercises.
Reduction of spasticity with repetitive transcranial magnetic stimulation in patients with spinal cord injury	[80]	14	C4-T12	C–D	2–17 months after SCI	DB	leg primary motor cortex	20 Hz	90% RMT	1600 × 5 (days)	baseline after the last sessions 1 week after the last rTMS session	MAS, VAS, SCI-SET, H_max_/M_max_, T-reflex, withdrawal reflex	HF-rTMS over the leg motor area can improve aspects of spasticity in patients with iSCI.
Repetitive transcranial magnetic stimulation on the modulation of cortical and spinal cord excitability in individuals with spinal cord injury	[81]	11					primary motor cortex	10 Hz, 1 Hz	90% RMT	7 days	baseline Immediately after, 30 and 60 min after rTMS	MEP, H-reflex, HD, MAS	HF-rTMS applied to M1 was able to promote increased cortical excitability in individuals with iSCI for at least 60 min; however, it did not modify spinal excitability or spasticity.

SCI, spinal cord injury; iSCI, incomplete SCI; rTMS, repetitive transcranial magnetic stimulation; HF-rTMS, high-frequency repetitive transcranial magnetic stimulation; AIS, American Spinal Injury Association impairment scale; DB, double-blinded; SB, single-blinded; RMT, resting motor threshold to TMS; AMT, active motor threshold to TMS; sEMG, surface electromyography; MEP, motor evoked potential; MAS, modified Ashworth scale; U&LEMS, upper and lower extremity motor scores; 10MWT, 10-meter walking test; JTT, Jebsen-Taylor hand function test; RTP, repetitive task practice; WISCI-II, walking index for SCI-II; TUG, timed up and go test; ARAT, action research arm test; EPT, electrical perceptual Test; MNCV, motor nerve conduction velocity; ADL, activity of daily living; SPPB, short physical performance battery; ABMS-2, ability for basic movement scale-2; FIM, functional independence measure; MVC, maximal voluntary contraction; 6MWT, 6-min walking test; SF-36, medical outcomes study 36-item short-form health survey; BWSTT, body weight-supported treadmill training; PPT, Purdue pegboard test; CMT, complete Minnesota test; TM, treadmill walking speed; VAS, visual analog scale; SCI-SET, spinal cord injury spasticity evaluation tool; Hmax/Mmax, ratio of maximum H-reflex to maximum M-wave; T-reflex, tendon reflex; HD, homosynaptic depression; M1, primary motor cortex; ^#^ Study combining rTMS with conventional rehabilitation therapy such as kinesiotherapy.

## Abbreviations

SCI	Spinal Cord Injury
TMS	Transcranial Magnetic Stimulation
rTMS	Repetitive Transcranial Magnetic Stimulation
iTBS	Intermittent Theta-Burst Stimulation
PAS	Paired Associative Stimulation
PNS	Peripheral Nerve Stimulation
tSCS	Transcutaneous Spinal Cord Stimulation
NPCs	Neural Progenitor Cells
LTP	Long-Term Potentiation
LTD	Long-Term Depression
NMDARs	N-Methyl-D-Aspartate Receptors
AMPARs	AMPA Receptors
iSCI	Incomplete Spinal Cord Injury
CST	Corticospinal Tract
sEMG	Surface Electromyography
MEP	Motor-Evoked Potential
9HPT	Nine-Hole Peg Test
HF-rTMS	High-Frequency Repetitive Transcranial Magnetic Stimulation
RGMa	Repulsive Guidance Molecule-a
CaMKII	Ca^2+^/Calmodulin-Dependent Kinase II
HPA	Hypothalamic Pituitary Adrenal
KCC2	Potassium-Chloride Cotransporter-2
GAD67	Glutamic Acid Decarboxylase 67
GABA	Gamma-Aminobutyric Acid
MAP2	Microtubule-Associated Protein 2
NGF	Nerve Growth Factor
BDNF	Brain-Derived Neurotrophic Factor
GAP-43	Growth-Associated Protein 43
PSD-95	Postsynaptic Densification Protein 95
MAP2K	Mitogen-Activated Protein Kinase Kinase
M1	Primary Motor Cortex
ASI	American Spinal Injury Association Impairment Scale
MVC	Maximal Voluntary Contraction
U&LEMS	Upper and Lower Extremity Motor Scores
10MWT	10-Meter Walking Test
WISCI-II	Walking Index for SCI-II
BWSTT	Body Weight-Supported Treadmill Training
MAS	Modified Ashworth Scale
VAS	Visual Analog Scale
SCI-SET	Spinal Cord Injury Spasticity Evaluation Tool
PMC	Premotor Cortex
fNIRS	Functional Near-Infrared Spectroscopy
DLPFC	Dorsolateral Prefrontal Cortex
NRS	Numeric Rating Scale
SF-MPQ2-CN	Short-Form McGill Pain Questionnaire-2 (Chinese Version)
cTBS	Continuous Theta-Burst Stimulation
BBB	Basso Beattie Bresnahan Scale
tsDCS	Transspinal Direct Current Stimulation
STDP	Spike Timing-Dependent Plasticity
PCMS	Paired Corticospinal–Motor Neuronal Stimulation
GRASSP	Graded and Redefined Assessment of Strength, Sensitivity, and Prehension
TEP	Transspinal Evoked Potential
rPMS	Repetitive peripheral magnetic stimulation
PAMS	Paired associative magnetic stimulation
MCAO	Middle Cerebral Artery Occlusion
